# Medical Reform

**Published:** 1847-09

**Authors:** 


### MEDICAL REFORM.

A cotemporary, in some strictures on the proceedings of the late National Medical Convention, condemns the preliminary education required in medical students, "as far in advance of the state of society as at present constituted in this country." This does not appear to us to have been the question before the Convention, or the one now before the profession. Is the standard erected by the Convention too high? That is the question to be settled, and we are free to confess that our answer is in the negative. It may be that students in every case will not be able at once to come up to it; but if we attempt no motion forward are we likely ever to progress? If nothing is said by those who feel the importance of a higher preliminary standard about an extension of preliminary studies, is there any hope that students will take the matter in hand and extend them? There must be agitation before there is reform. We have no hope of an immediate acquiescence in the suggestions of the convention, but we feel quite sure that the discussion of the various topics started by it will lead to important reforms.

One sentiment advanced by our cotemporary strikes us as curious, coming as it does from a public teacher of medicine. It is the following: "A physician must converse with those who employ him upon those subjects adapted to their knowledge and tastes; and if he has been educated above their standard he must bring himself down to their level; and the inevitable consequence is, that he forgets all that he is not in the habit of daily using, and in a few years is but little elevated above his neighbors." Now, we had supposed the operation of knowledge in such cases was the reverse of this, and that instead of being degraded and extinguished, its tendency was to elevate, ennoble, and refine those who were brought within its influence. We had thought that the educated physician was likely to instruct his less fortunate associates, and to act as a leaven in the community where he resided, exciting in the whole mass better tastes and more generous impulses. We shall be surprised if our country brethren do not take this remark of our cotemporary as anything but complimentary to them. They will be slow to admit, we think, that, every year since they received their diplomas, they have been multiplying the fathoms of descent towards the level of their ignorant neighbors.

The writer to whom we have been referring admits the value of "*Clinical instruction*," but insists on the impossibility of imparting it in many of the schools. Upon this admission one of his critics in a Philadelphia journal has the following rather stringent suggestion:

> "It is respectfully submitted that a school which proposes to educate *practitioners* of medicine, and cannot illustrate pathology with living cases, is bound either to close its doors, or to announce that patients who employ its graduates must do so at their proper peril, regarding the title of doctor conferred by it as indicating no competency to treat disease."

